# The ability of different compositions of calcium silicate and epoxy sealers to withstand gutta percha removal via in vitro pull-out testing

**DOI:** 10.1038/s41405-024-00212-9

**Published:** 2024-04-08

**Authors:** Idan Stiklaru, Ella Lalum, Sobhi Hamoud, Maayan Paz, Avi Levin, Joe Ben Itzhak, Nirit Yavnai, Pavel Gorenbein, Michael Solomonov

**Affiliations:** 1 Department of Endodontics, Israel Defense Forces (IDF), Medical Corps, Tel Hashomer, Shiba Road 2, Ramat- gan, Israel; 2 ”Bina” Program, Faculty of Dental Medicine, Hebrew University of Jerusalem, 12271 Jerusalem, Israel; 3 Department of Community Dentistry, Faculty of Dental Medicine, Hebrew University, Hadassah Ein Kerem Campus, 12271 Jerusalem, Israel; 4 Medical Supplies, Pharmacy, and Biomedical Engineering Branch, Israel Defense Forces (IDF), Medical Corps, Tel Aviv, Israel

**Keywords:** Root canal treatment, Endodontic instruments

## Abstract

**Objective:**

examination of the influence of chemical composition changes on the ability of sealers to withstand a pull-out test.

**Materials and methods:**

Fifty distal or palatal canals of extracted teeth were prepared by Dc Taper files. The teeth were divided into five groups: AH Plus, BJM RCS, Total Fill BC,AH Plus Bioceramic and a group with Gutta Percha with no sealer added. Ten days after obturation, each cone was subjected to the “pull-out test” with the Shimadzo Universal Testing Machine until it was torn or removed from the canal. A force to Stroke graph was generated and the maximum vertex of this graph was recorded. The number of times the cone was torn or removed was recorded.

**Results:**

The amount of force needed to remove or rupture the cone was significantly higher in all sealer groups compared to the AH Plus Bioceramic group. The force needed for the AH Plus group was double that needed for the AH Plus Bioceramic group 4 (1.87 ± 0.53 N vs 0.93 ± 0.48 N, respectively, *P* < 0.001). All of the cones (*n* = 10) in the AH Plus Bio Ceramic Sealer group were removed in their entirety (*P* = 0.01 compared to each of the other groups).

**Conclusions:**

The addition of macromolecules to epoxy sealer does not change the material’s ability to withstand the pull-out test. Decreasing the amount of tri- and di-calcium silicate compounds combined with increasing amounts of zirconium oxide in a Bioceramic sealer significantly decreased the material’s ability to withstand the pull-out test.

## Objective

Sealers comprise an essential part of the process of obturation [1]. For the past few decades, Epoxy Sealer AH Plus has been essentially considered the gold standard among the commercially available endodontic sealers [2]. Sealing properties of epoxy sealers are dependent mainly upon their adhesion ability [3]. The BJM Root Canal Sealer (BJM Laboratories, Or-Yehuda, Israel) is a recently introduced epoxy-based sealer with the addition of anti-biofilm macromolecules. It contains 1.6–3.3% wt of antibiofilm molecules- named “BioSafe” [4].

Tricalcium silicate-based (TCS) cements have gained popularity due to their biological properties such as biocompatibility and ease of handling for dentists [5]. They afford sealing capability also by means of expansion during setting [5].

By using tricalcium silicate cement instead of Portland cement found in MTA, these materials address concerns of leaching trace elements and aluminum. Bismuth oxide is replaced by tantalum oxide in paste and putty, and zirconium oxide in the sealer, addressing major MTA concerns. Handling issues are resolved with bioceramics available in putty and paste forms, and the sealer is pre-mixed for convenience. The chemical formulation includes a second cementitious phase—calcium phosphate monobasic—to promote biomineralization. The presence of phosphate enhances biomineralization, making bioceramics suitable for this purpose [6].

According to the manufacture and recent publication [7], the newly launched AH Plus Bioceramic Sealer (Dentsply Sirona, York, PA) contains a relatively low volume of tricalcium silicate (5–15% wt) and a high volume of zirconium dioxide (50–70% wt).

The most researched material among the currently commercially available TCS is the Total Fill BC Sealer (FKG Dentaire SA, La Chaux-de-Fonds, Switzerland), which contains a comparatively higher volume of tricalcium silicate (20–35% wt) as well as dicalcium silicate (7–15% wt), with a much lower volume of zirconium oxide (35–45% wt).

To the best of the authors’ knowledge, there are no data on the consequences of changing the chemical composition of these sealers, such as the addition of macromolecules to epoxy-based sealers or changes of the main component volume in TCS on the properties of the materials. An in vitro pull-out test can reportedly serve as the initial evaluation of sealing ability of various sealers, cements and sealing mechanisms [8–10].

A recent micro-ct analysis [11] aimed to assess adaptation of epoxy sealer and a TCS sealer to GP cone by means of gap formation along the specimens. The authors concluded that while no specimens exhibited a completely gap-free area along the entire interface of GP-sealer, oval canals filled with AH Plus demonstrated fewer gaps compared to those filled with EndoSequence BC Sealer.

The aim of the current study, therefore, is to examine the influence of chemical composition changes on the ability of sealers to withstand GP cone removal utilizing pull-out testing in vitro, which is an indirect method to test their sealing ability.

## Materials and Methods

The study was conducted in accordance with the Declaration of Helsinki and was approved by the Institutional Review Board. Approval number: #2243-2021 (04/21).

### Teeth

Fifty extracted mandibular and maxillary molars with relatively similar distal (for mandibular teeth) or palatal (for maxillary teeth) canals (as was inspected utilizing 2 angles of x-ray images per tooth) and with complete root formation, angle of curvature close to zero and initial canal diameter less than N20 K-flle (Dentsply Sirona, York, PA) were collected. This sample size was chosen in accordance with recently published in-vitro endodontic sealer examinations [12–14] and after calculations with Winpepi version 11.65 software, with a significance level of alpha = 0.05. The teeth were stored in 0.9% NaCl solution. They were sectioned by a 0.3 mm thick Horico separation disc at the cementoenamel junction (CEJ) in order to create better and easier access and visibility. The canals were prepared to an ISO 45 by means of DC Taper rotary files (SS White Dental Inc., Lakewood, NJ, USA) to a length of 8 mm from the CEJ (in order to eliminate possible differences in the middle and apical curvatures and to create a standardized length and diameter). Irrigation was with 5 ml 3% NaOCl per canal, and the final irrigation was with 2 ml 17% EDTA solution (Meta- Biomed Chungcheongbuk-do, Korea)Prior to obturation the teeth were dried using paper points (Dentsply Sirona, York, PA).

**Fig. 1:**
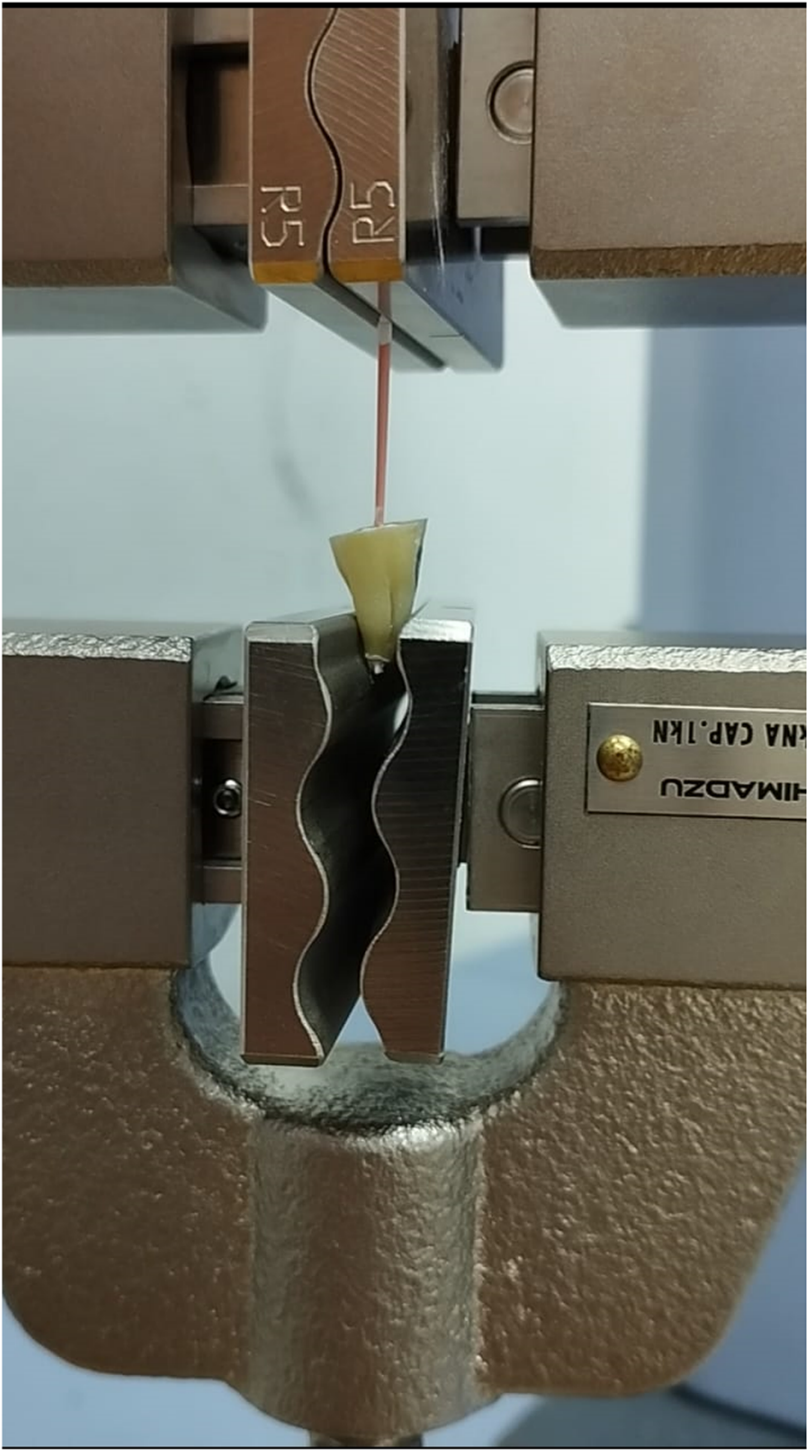
The testing apparatus. Gutta Percha cone being pulled by the upper forceps while the tooth is held by the lower forceps.

The teeth were then distributed into four sealer groups: 1. AH Plus, 2. BJM RCS, Total Fill BC and AH Plus Bioceramic. And a fifth group obturated solely with gutta percha (GP, Dentsply Sirona, York, PA). The canals were obturated by the “single cone” technique consisting of a 45 ISO GP cone with a dedicated sealer in each group. The GP cones were left uncut coronally to the orifices. Another two teeth per group served as control: Their canals were prepared as mentioned above and then they were “sealed” using only the sealer with no GP, in order to verify setting of the sealer prior to commencing the pull- out test. The teeth were kept in 100% humidity and at a temperature of 37 °C for 10 days, after which they were subjected to the “pull-out test” by means of the Shimadzo Universal Testing Machine: the teeth were held by the lower forceps and the Gutta Percha cone was held and pulled at a speed of 1 mm\min by the upper forceps until the cone was torn or removed entirely from the canal (Fig. 1). A force (in Newtons) to stroke (in millimeters) graph was generated for each specimen and the maximum vertex of this graph (i.e., the force at which the cone was torn or removed) was recorded. The number of times the cone was torn or removed was recorded for each group. The test was conducted by one author (I.S.) who was blinded to the type of sealer used in each group.

### Statistical analysis

All statistics were performed using SPSS software version 27 (IBM, North Castle, NY, USA).

Continuous variables were checked for their distribution and are presented by means ± standard deviation. The force to pull out the Gutta Percha which was measured by Newtons was found normally distributed (one way Kolmogorov Smirnov test, *p* = 0.085). Categorical variables are presented by percentages. Comparisons of means were analyzed with the one-way ANOVA test, and the post hoc results by Bonferroni tests. Associations between distributions were performed with a chi-square test. Differences with *P* < 0.05 were considered statistically significant.

## Results

The amount of force needed to remove or rupture the GP cone was significantly higher in groups 1-3 compered to group 4 (AH plus Bioceramic Sealer). The force needed for group 1 (AH plus epoxy cement) was double the one needed for group 4 (1.87 ± 0.53 N compared to 0.93 ± 0.48 N, respectively, *p* < 0.001), and even more than double for group 2 (BJM RCS) (2.05 ± 0.47 N compared to 0.93 ± 0.48 N, respectively, p < 0.001). The force needed by group 3 (Total Fill BC sealer) was lower (1.63 ± 0.32 N) but not significantly less than that needed by the epoxy sealers (*p* = 0.010) although significantly higher (*p* < 0.001) than the force needed for group 4. These results are presented in Table 1.

**Table 1:** The force (in Newtons) for gutta percha cone removal or rupture in each sealer group.

	1 (AH Plus)	2 (BJM RCS)	3 (Total Fill BS)	4 (AH Plus Bioceramic)
1	1.3926	2.8198	1.86244	0.7882
2	2.7604	2.6075	2.0918	0.6545
3	2.3175	2.1536	1.3621	0.2127
4	0.9171	2.0189	1.4056	0.5133
5	1.4601	1.6676	1.7335	1.6941
6	1.7754	1.7252	1.6778	0.7954
7	2.1181	2.3712	0.9941	1.2243
8	1.7592	2.1331	1.7948	0.8051
9	2.0226	1.2329	1.8732	1.7131
10	2.1754	1.7605	1.5184	0.9285
Mean ± SD	1.86984 ± 0.53	2.04903 ± 0.47	1.631374 ± 0.32	0.93292 ± 0.48

Group	Compared to	95% Confidence interval	*p*-value*
1	2	−0.75to 0.39	1.00
	3	−0.33 to 0.81	1.00
	4	−0.36 to 1.51	**<** **0.001**
2	3	−0.15 to 0.99	0.291
	4	0.55–1.68	**<** **0.001**
3	4	0.13–1.27	**0.01**

Group 1 = AH Plus.

Group 2 = BJM RCS.

Group 3= Total Fill BS.

Group 4 = AH Plus Bioceramic.

*One-way ANOVA with Bonferroni post hoc tests.

Bold indicates significant.

All of the cones (*n* = 10) in group 4 (AH Plus Bio Ceramic Sealer) were removed in their entirety, and this result was significantly different compared to each of the other three sealer groups (*p* = 0.01), and to all three sealer groups in combination (p < 0.001). There was no significant difference between the other three sealer groups. The number of times the cone was removed or torn for each group is listed in Table 2.

**Table 2:** The number of times the cone was ruptured or completely removed for each group.

	AH Plus	BJM	Total Fill	AH Plus Bioceramic
1	Torn	Torn	Removed	Removed
2	Torn	Torn	Torn	Removed
3	Torn	Torn	Torn	Removed
4	Torn	Torn	Removed	Removed
5	Removed	Removed	Removed	Removed
6	Torn	Torn	Removed	Removed
7	Torn	Torn	Removed	Removed
8	Torn	Torn	Torn	Removed
9	Torn	Removed	Torn	Removed
10	Removed	Removed	Torn	Removed
Total Removed	2	3	5	10

Group	Compared to	*p* value*
1 (AH Plus)	2	1.000
	3	0.170
	4	**<** **0.01**
2 (BJM RCS)	3	0.370
	4	**0.003**
3 (Total Fill BS)	4	**0.033**

Group 1 = AH Plus.

Group 2 = BJM RCS.

Group 3= Total Fill BS.

Group 4 = AH Plus Bioceramic.

Chi square test*.

Bold indicates significant.

As for the fifth, GP group- the force needed to remove the cone in all of the teeth was zero Newtons, and it seems that there is no tensile strength for the GP cone itself.

## Discussion

A multitude of various types of root canal sealers have been developed over the years. The sealing can be achieved by a number of different mechanisms: epoxy sealers work by means of adhesion to the canal wall and the GP cone, while calcium silicate-based sealers (CSBS) group can also employ expansion. The pull-out test, as suggested in previous studies [8–10], can provide quantification of both mechanisms. It seems that the addition of “BioSafe” to an epoxy sealer in relatively small amounts (1.6–3.3% wt) does not affect the adhesion mechanism of the epoxy sealers as was assessed by means of pull- out testing. TCS, often referred to as “Bioceramic” sealers, has gained widespread popularity, and numerous brands and materials have appeared on the market [5], all claiming to be tricalcium silicate cements, although not all of the companies provide the precise material composition of their product.

A recently developed AH Plus Bioceramic Sealer (Dentsply Sirona) contains a relatively low volume of tricalcium silicate (as low as 5–15% wt) and a high volume of zirconium dioxide (50–70% wt). This composition is quite different compared to the Total Fill BC Sealer) the most researched Bioceramic sealer to date. Specifically, the Total Fill BC Sealer contains higher volumes of tricalcium silicate (20–35% wt) and dicalcium silicate (7-15% wt) and much less (35–45% wt) zirconium oxide. Most of the properties of the tricalcium silicate cement sealer group are dependent upon the tri- and di-calcium silicates, and apparently on their relative volume in the material as well [12, 15]. It follows that lowering the amount of this main component of this material may significantly alter its properties. Zirconium oxide is used solely to perform as a radio pacifier [13], and increasing the volume of the zirconium in a sealer might not to be justified.

The results of the current study demonstrated a substantial difference in pull-out strength (in Newtons) between the novel AH Plus Bioceramic Sealer compared to the three other tested sealers, while the latter showed slight differences between them, none reaching a level of significance. One possible explanation for this finding is that lowering tri- and di-calcium silicate levels decreases the expansion ability of TCS. A second option is that the change in sealer composition might have increased the gaps formation in the interphase GP cone – sealer, as was demonstrated by a recent work [11].

All of the GP cones in the AH Plus Bioceramic sealer group were removed from the sealer and exited the canal in their entirety with a significantly lower force compared to the other three sealer groups, this result emphasizes the lower adaptation of the AH Plus Bioceramic sealer to the gutta percha cone, although removed cones were encountered in them as well. The force needed to remove the cone by those sealers was similar to the one needed to tear the cone but nevertheless significantly higher than the force needed by the AH Plus Bioceramic sealer. In the fifth group of ten teeth with solely gutta percha cones the force needed for the removal of the cones was zero. Thus, all of the results in the other groups can be related to the sealers ability to withstand the pull-out testing, rather than the friction of the cone itself. As was demonstrated in the results of the current study, it can be assumed the decreasing of the amount of tri- and di-calcium silicate compounds combined with increasing amounts of zirconium oxide in a Bioceramic sealer significantly decreased the material’s ability to withstand the pull-out test.

Possible explanations for the difference between torn and removed GP include: 1. unpredictable width of the sealer layer in the one-cone technique because of the non-round cross-section of the canals [14]; 2. the teeth were extracted from different age groups of patients, which can affect the diameter and quantity of the dentinal tubules, the mineral content of the dentin [16]; 3. there are no data on the pulp condition prior to extraction, and biofilm remnants can modify the sealer’s abilities [17, 18].

## Conclusions

The addition of the BioSafe macromolecule to epoxy sealer does not change the material’s ability to withstand the pull-out test. AH Plus Bioceramic Sealer attachment to the root canal wall was lower than any of the other sealers tested.

## Data Availability

The datasets used and/or analyzed during the current study are available from the corresponding author on reasonable request.
